# Fluid Intake and Decreased Risk for Hospitalization for Dengue Fever, Nicaragua

**DOI:** 10.3201/eid0908.020456

**Published:** 2003-08

**Authors:** Eva Harris, Leonel Pérez, Christina R. Phares, Maria de los Angeles Pérez, Wendy Idiaquez, Julio Rocha, Ricardo Cuadra, Emelina Hernandez, Luisa Amanda Campos, Alcides Gonzalez, Juan Jose Amador, Angel Balmaseda

**Affiliations:** * University of California, Berkeley, California, USA; † Ministerio de Salud, Managua, Nicaragua; ‡ Hospital Infantil Manuel de Jesús Rivera, Managua, Nicaragua; § Hospital Escuela Oscar Danilo Rosales Argüello, Leon, Nicaragua; ¶ Centro de Salud Francisco Buitrago, Managua, Nicaragua

**Keywords:** dengue, hospitalization, fluid intake, Nicaragua, dispatch

## Abstract

In a hospital and health center-based study in Nicaragua, fluid intake during the 24 hours before being seen by a clinician was statistically associated with decreased risk for hospitalization of dengue fever patients. Similar results were obtained for children <15 years of age and older adolescents and adults in independent analyses.

Dengue fever *(*DF) and dengue hemorrhagic fever/dengue shock syndrome *(*DHF/DSS) are major public health problems worldwide (*1*). In addition to causing considerable illness and death, dengue epidemics also have a major economic impact, attributable primarily to the cost of medical care, plus vector control programs and lost productivity (*2*,*3*). DF and DHF/DSS are indistinguishable in the initial febrile phase, and few reliable clinical prognostic indicators of DHF/DSS exist (*4*). Near the time of defervescence, the plasma leakage syndrome of DHF/DSS can develop suddenly and be fatal if not managed properly. As a result, in areas relatively new to dengue, such as Latin America where DF and DHF/DSS have been spreading over the last 2 decades, high hospitalization rates for DF can occur because of the fear of discharging a patient whose case may progress to DHF/DSS. As DHF/DSS becomes hyperendemic, as in Southeast Asia, and extensive experience is garnered in case management, most hospitalizations for dengue are due to frank DHF/DSS. We conducted a study to assess the extent of hospitalization attributable to dengue in Nicaragua and to examine the role of factors such as prior fluid intake on the risk for hospitalization.

## The Study

Participating hospitals and health centers in urban centers of the Pacific region of Nicaragua included the Hospital Escuela Oscar Danilo Rosales Argüello in León, the pediatric reference Hospital Infantil Manuel de Jesus Rivera in Managua, and four health centers in Managua, which serve a combined population of 1.95 million people. A cross-sectional study of 2,820 suspected dengue cases was conducted from January 1 to December 31, 1999. Enrollment criteria consisted of acute febrile illness accompanied by two or more of the following symptoms of DF: fever, severe headache, retroorbital pain, myalgias, arthralgias, and rash (*4*,*5*). A standardized questionnaire was used to collect demographic and clinical information, including ingestion of fluids in the 24 hours before first being seen by a clinician, as reported by the patient or parent. Of the 1,312 patients who tested positive for DENV infection, the mean age was 15.3 years *(*range 0–85), and 557 *(*42%) were male *(*Table 1). Venous blood was drawn at the first visit, and convalescent-phase serum specimens were obtained when possible *(*19% of patients). Clinical evolution of the illness among hospitalized patients was documented by chart review with standardized data-entry forms. This study was approved by the Institutional Review Boards of both University of California, Berkeley *(*#99-4-38) and the Centro Nacional de Diagnóstico y Referencia of the Nicaraguan Ministry of Health *(*#99-04).

**Table 1:** Characteristics of all hospitalized and unhospitalized dengue patients^a^

Data	Hospitalized cases			Unhospitalized cases		Total		
	n		Mean *(*sd) or % of group	n	Mean *(*sd) or % of group	n	Mean *(*sd) or % of group	
**Demographic data**								
Age *(*y)	478		9.4 *(*8.9)	834	18.7 *(*15.5)	1,312	15.3 *(*14.2)	
Male	229		47.8%	328	39.2%	557	42.3%	
**Distance *(*km) to health facility**	464		10.0 *(*25.0)	831	2.7 *(*6.1)	1,295	5.3 *(*16.1)	
Disease classification**^a^**								
Classic DF	113		23.5%	706	84.4%	819	62.1%	
DFHem	240		50.0%	129	15.4%	369	28.0%	
DHF	67		14.0%	0	0%	67	5.1%	
DSS	16		3.3%	1	0.1%	17	1.3%	
DSAS	30		6.3%	0	0%	30	2.3%	
No classification	14		2.9%	1	0.1%	15	1.1%	
*Duration of hospitalization (days)^b^*								
***Classic DF***	52		5.7 *(*1.7)					
***DFHem***	95		5.1 *(*1.7)					
***DHF***	56		6.1^c^ *(*1.5)					
***DSS***	15		6.9^c,d^ *(*1.9)					
***DSAS***	28		6.1^c^ *(*1.8)					
No classification	11	6.2 *(*1.9)						
**Clinical data at presentation**								
No. of glasses of fluid ingested during previous 24 h^e^	331		2.9 *(*2.3)	757	5.6 *(*3.9)	1,088	4.8 *(*3.7)	
Thrombocytopenia	289		60.2%	58	6.9%	347	26.4%	
Anorexia	239		50.9%	422	53.3%	661	52.4%	
Stomach pain	271		58.4%	404	51.5%	675	54.4%	
Days since onset of symptoms	476		5.5 *(*5.6)	826	5.2 *(*5.7)	1,302	5.3 *(*5.7)	

^a^DF, dengue fever; DFHem, DF with hemorrhagic manifestations; DHF, dengue hemorrhagic fever; DSS, dengue shock syndrome; DSAS, dengue with signs associated with shock. ^b^Information on the duration of hospitalization was available from 52 *(*46%) of hospitalized classic DF patients; 95 *(*40%) of hospitalized DFHem patients; 56 *(*84%) of hospitalized DHF patients; 15 *(*94%) of hospitalized DSS patients; and 28 *(*93%) of hospitalized DSAS patients. Outliers *(*>12 days) were removed before analysis. ^c^The mean duration of hospitalization was significantly longer for DHF cases, DSS cases, and DSAS cases as compared with duration for DFHem cases *(*p<0.05 for each, Student t test). ^d^The mean duration of hospitalization was significantly longer for DSS cases compared with duration for classic DF cases *(*p<0.05, Student t test). ^e^The average glass contains approximately 8 oz.

Dengue virus *(*DENV) infection was identified in 1,312 patients by serologic methods *(*immunoglobulin M-capture enzyme-linked immunosorbent assay [ELISA] or inhibition ELISA for total anti-DENV antibodies), virologic means *(*virus isolation, reverse transcriptase-polymerase chain reaction), or both (*5*). DENV2, DENV4, and DENV3 accounted for 79%, 17%, and 5% of the typed viruses, respectively. Dengue fever was divided into classic DF and DF with hemorrhagic manifestations *(*DFHem); severe dengue was defined as DHF *(*World Health Organization classification DHF grades I and II), DSS *(*World Health Organization DHF grades III and IV) (*1*), or an additional classification designated “dengue with signs associated with shock” *(*DSAS). DSAS was designated when either hypotension for age or narrow pulse pressure plus clinical signs of shock were present and serial hematocrit and platelet counts failed to document thrombocytopenia and hemoconcentration, potentially attributable to fluid replacement therapy (*5*). The relationship between hospitalization and fluid intake was assessed by unconditional logistic regression. Because virtually every patient with severe dengue was hospitalized, such risk factor analysis was not suitable for these patients as a separate group. Thus, the analysis was restricted to DF and DFHem patients. Univariate and multivariate analyses were performed separately on children *(*<15 years of age) and older adolescents and adults *(*>15 years of age). Covariates found to be significant in univariate analyses were included in the initial multivariate models *(*Table 2).

**Table 2:** Crude and adjusted odds ratios and 95% confidence intervals for factors potentially associated with hospitalization for classic dengue fever or dengue fever with hemorrhagic manifestations

	Children *(*<15 years of age)^a^			Older adolescents and adults *(*>15 years of age)^a^		
Characteristic	No. of patients^b^	OR *(*95% CI)^c^ Crude	OR *(*95% CI)^c^ Adjusted^d^	# of patients^b^	OR *(*95% CI)^c^ Crude	OR *(*95% CI)^c^ Adjusted^f^
**Fluid intake during 24-h period before presentation**	587			405		
For each additional glass		0.68 *(*0.62 to 0.75)	0.74 *(*0.66 to 0.83)		0.67 *(*0.5 to 0.79)	0.71 *(*0.59 to 0.85)
>5 glasses		0.14 *(*0.08 to 0.25)	0.19 *(*0.09 to 0.39)^e^		0.16 *(*0.06 to 0.43)	0.20 *(*0.07 to 0.57)^g^
*Age*	719		—^h^	464		—
For each additional year		0.93 *(*0.8 to 0.97)			0.98 *(*0.96 to 1.00)	
**Sex**	718			464		
Male	395	1.43 *(*1.06 to 1.94)		291	1.27 *(*0.74 to 2.17)	
Female	323			173		
**Distance from healthcare facility**	701			460		
For each additional 5 km		2.13 *(*1.68 to 2.69)	1.46 *(*1.12 to 1.91)		1.16 *(*0.92 to 1.46)	—
**Date of onset of symptoms**	709			455		
For each additional month		1.26 *(*1.16 to 1.37)	1.51 *(*1.26 to 1.81)		1.87 *(*1.53 to 2.29)	2.08 *(*1.53 to 2.83)
**Days between onset of symptoms and being seen at facility**	713			457		
For each additional day		1.04 *(*1.0 to 1.07)	—		0.98 *(*0.93 to 1.03)	—
**Thrombocytopenia**	499			227		
Yes	189	6.5 *(*4.25 to 9.96)	6.16 *(*3.57 to 10.64)	33	3.31 *(*1.53 to 7.15)	3.62 *(*1.24 to 10.52)
No	310			194		
**Stomach pain**	681			439		
Yes	370	0.94 *(*0.69 to 1.28)	—	216	1.50 *(*0.89 to 2.56)	—
No	311			223		

^a^The age distribution of children <15 years of age was 7.2 *(*SD 3.9) with a range from 0 to 14 years and that of older adolescents and adults was 30.6 *(*SD 13.9) with a range of 15 to 85 years. ^b^The numbers differ based on completeness of information for each variable. ^c^OR, odds ratios; CI, 95% confidence intervals. ^d^Adjusted for glasses of liquid consumed *(*continuous variable), distance from healthcare facility, date of onset of symptoms, and thrombocytopenia. ^e^The adjusted OR and 95% CI for glasses of liquid consumed *(*dichotomous variable) were obtained from a separate model that adjusted for the same factors as footnote d. ^f^Adjusted for glasses of liquid consumed *(*continuous variable), date of onset of symptoms, and thrombocytopenia. ^g^The adjusted OR and 95% CI for glasses of liquid consumed *(*dichotomous variable) were obtained from a separate model that adjusted for the same factors as footnote f. ^h^ Dash indicates that this variable did not significantly contribute to the multivariate model.

Although a much higher proportion of severe dengue patients were hospitalized compared with DF and DFHem patients, larger absolute numbers of hospitalizations were due to classic DF and DFHem because many more cases of DF/DFHem than of severe dengue occur *(*Table 1). Thus, of all the laboratory-confirmed DENV-positive hospitalized patients, classic DF *(*23.5%) and DFHem *(*50.0%) cases comprised three times as many as DHF, DSS, and DSAS combined *(*23.6%). These profiles have been maintained from year to year, with 28% and 20% of hospitalized cases due to classic DF and 51% and 58% due to DFHem in 1998 and 2000, respectively (*5*). Furthermore, the mean duration of hospitalization was similar for patients with DF and those with severe dengue *(*Table 1).

## Conclusions

Among children with DF or DFHem, ingestion of fluid in the 24 hours before being seen by a clinician was found to be protective against hospitalization after adjusting for distance from health facility, date of symptom onset, and thrombocytopenia *(*odds ratio [OR]=0.74 per each additional glass consumed, 95% confidence intervals [CI] 0.66 to 0.83, p<0.01) *(*Table 2). Similar results were obtained for older adolescents and adults after adjusting for date of symptom onset and thrombocytopenia, with an OR of 0.71 *(*95% CI 0.59 to 0.85, p<0.01). In analyses with a statistical model that did not assume a linear relationship between number of glasses ingested and hospitalization, additional benefit was noted for each glass up to five, after which the incremental benefit remained constant. Thus, in a model that compared the fluid intake of five glasses or less than five glasses with ingestion of more than five glasses, the adjusted OR for hospitalization was 0.19 *(*95% CI 0.09 to 0.39, p<0.01) among DF and DFHem cases in children and 0.20 *(*95% CI 0.07 to 0.57, p<0.01) in those >15 years of age *(*Table 2). We also performed the analysis with all disease states, including severe dengue cases, and the results were virtually identical to those obtained with only DF/DFHem cases *(*data not shown).

The most common liquid ingested was water *(*70%), followed by fruit juice *(*42%), lemonade *(*27%), milk *(*25%), coffee *(*14%), oral dehydration serum *(*6%), and tea *(*2%). The mean number of glasses ingested by nonhospitalized DF/DFHem case-patients was 5.2, whereas the mean number glasses ingested by hospitalized DF/DFHem patients was 2.8, similar to the mean of 2.9 glasses ingested by hospitalized severe patients *(*DHF/DSS/DSAS). These findings suggest that maintaining hydration may lead to reduced hospitalizations of patients with DF/DFHem. Other independent risk factors for hospitalization included in the final multivariate model were increasing distance from the healthcare facility, later date of symptom onset, and the presence of thrombocytopenia. The risk conferred by later date of symptom onset reflects increased awareness of dengue by medical staff as the annual epidemic intensified. Because thrombocytopenia is an indication for hospitalization *(*especially for pediatric dengue patients), its emergence as a risk factor is not surprising.

Because inpatient medical care of DF and DFHem patients can contribute significantly to the economic impact of dengue, we sought to define outpatient measures that could decrease DF hospitalization rates. Dengue patients are likely to be susceptible to dehydration because of high fever and concomitant anorexia. While oral rehydration therapy is listed as standard outpatient management for DHF (*1*), little discussion exists on the use of such therapy for DF and DFHem cases (*4*), and no published studies have examined the effect of fluid intake at home. Our results show that this simple measure may have a significant protective effect against hospitalization and potentially on the severity of DF/DFHem, although causality cannot be confirmed because of the observational nature of this study. To definitively demonstrate this effect, this question must be investigated by prospective intervention studies. However, our data do suggest that promoting a high fluid intake at home could help reduce the need for hospitalization and thus attenuate economic impact of dengue in countries experiencing epidemics of dengue fever.
